# Diseases of the Skin

**Published:** 1898-10

**Authors:** 


### BOOK NOTICES.

Diseases of the Skin: Their Constitutional Nature and Cure. By J. Compton Burnett, M. D. Third edition revised and enlarged. Philadelphia: Bœricke & Tafel. 1898. Price, cloth, $1.00 net. By mail, $1.07.

This little monograph has been introduced to the profession before. It is the third edition.

Its pages are a remarkable revelation to the world of the powers of Homœopathy in the treatment of skin diseases.

In the preface the author gives his views as to the relationship of the skin to the general economy.

He says: "I do not maintain that there is no such thing as a skin disease of a purely local nature, such as common phtheiriasis and other parasitic dirt-diseases that impinge upon the skin, but speaking generally, I do maintain the following points: First, that the skin is a very important *living* organ of the body. Second, that it stands in intimate, though ill-understood relationship to *all* the internal organs and parts. Third, that its healthiness is conditioned by the general healthiness of the organism—*i. e.*, a healthy skin on an unhealthy body is inconceivable. Fourth, that speaking generally, its unhealthiness—its diseases—come from within, sometimes even when they initially impinge upon it from without. Fifth, that being *biologically within* the organism, being *fed from within*, having its life from within, having its health from within, and having its diseases from within, it must also be treated medicinally from within. Sixth, that skin diseases are most commonly not merely organic, but at the same time organismic, or constitutional. Seventh, that the skin being an excretory organ, and being spread out all over the organism, is often made use of by nature to keep the internal organs free from disease. Eighth, that as each portion of the skin corresponds vitally with some internal organ or part, so the skin disease is often merely the outward expression of internal disease. Ninth, that in fine, the generally received external treatment of diseases of the skin, whether with lotions or ointments, or whatsoever else, is demonstrably shallow in conception, wrong in theory, harmful in practice, and therefore inadvisable."

In this exposition of the author's views the reader will perceive the true homœopathic character. It may be added that the author defiantly claims that these views are "absolutely irrefutable."

He then proceeds to recount cases that illustrate the truth of his views.

Thus he tells of various diseases spontaneously cured by attacks of erysipelas. On the other hand, he gives instances of diseases of a serious character produced by the suppression of skin eruptions. He gives a striking case of cataract of both eyes following upon the artificial suppression of ringworm.

A case of cataract was produced by the suppression of scabies. Dr. Burnett cured the cataract by giving Sulphur in potency. As soon as the cataract disappeared "a number of furuncles appeared on the arms."

The author thinks that sties are due to the effects of vaccination.

A most instructive essay upon lupus is given, in which the author maintains the constitutional nature of lupus, and quotes the results of its examination by a great number of investigators, all pointing to the same conclusion.

At page 97 the author asserts that his whole object in writing the book is to show "that diseases of the skin are really diseases of the system, though *on* the skin." He hopes that the time will come when we shall, perhaps, be able to see why certain cutaneous diseases affect certain parts preferentially, and also why when these diseases are driven in whence they came, by external means, certain internal organs have to bear the brunt of it.

This book is a great encouragement to all who seek to treat skin diseases homœopathically, and is indeed a guide in managing them.

